# Una nueva etapa para Advances in Laboratory Medicine/Avances en Medicina de Laboratorio

**DOI:** 10.1515/almed-2023-0113

**Published:** 2023-09-08

**Authors:** Álvaro González, Roser Ferrer-Costa, Pilar Fernández-Calle, Ignacio Arribas, Wladimiro Jiménez

**Affiliations:** Servicio de Bioquímica, Clínica Universidad de Navarra, Navarra, España; Servicio de Bioquímica, Hospital Universitario Vall d’Hebron, Barcelona, España; Servicio de Bioquímica, Hospital Universitario La Paz, Madrid, España; Servicio de Bioquímica, Hospital Universitario Ramón y Cajal, Madrid, España; Servicio de Bioquímica y Genética Molecular, Hospital Clinic Universitari de Barcelona, Barcelona, España

**Keywords:** PubMed, Scopus, Journal Citation Reports, Factor de Impacto, SCImago

Dentro del Editorial que acompañaba al primer número de Adv. Lab. Med., en marzo de 2020 se describían algunos de los objetivos con los que nacía la revista, y entre ellos estaba ocupar una presencia significativa en los índices de factores de impacto [1]. Un primer paso necesario era que la revista fuera incluida en los motores de búsqueda de las bases de datos biomédicas más relevantes, ya que en caso contrario la difusión quedaba limitada a una comunidad relativamente pequeña de lectores. Hoy en día la revista ya se encuentra incorporada en las más importantes bases de datos de consulta en el área biomédica como son PubMed Central (de National Center for Biothecnology Information, NCBI), Web of Science (de Clarivate Analytics) y Scopus (de Elsevier). Con ello se ha conseguido que los trabajos publicados sean visibles a toda la comunidad científica, que desde estos motores disponen directamente del acceso abierto a los artículos. No obstante, es interesante señalar que antes de aparecer en estas bases de datos, Adv. Lab. Med. ya contaba con un número relevante de accesos y de citas, lo que subraya su calidad y el interés por los artículos aquí publicados. Entre ellos están los originales, revisiones y los trabajos y recomendaciones científicas enviados por Comisiones de la Sociedad Española de Medicina de Laboratorio.

La medida de la calidad y prestigio de una revista científica se basa en una serie de índices bibliométricos cuantitativos que además señalan la dificultad de publicar en ella. Probablemente el indicador bibliométrico más usado es el factor de impacto (FI) que publica anualmente Journal Citation Reports (de Clarivate Analytics) a partir de la información de Web of Science. Este índice se calcula como la media de citas recibidas por artículo publicado en la revista durante los dos años anteriores [2]. Así el FI más reciente, de 2022, sería el número de citas en 2022 de artículos publicados en 2021 y 2020 dividido por el número de artículos en 2021 y 2020). Otro indicador bibliométrico importante es la posición de la revista dentro de su grupo, que en el caso de Adv. Lab. Med. sería Medical Laboratory Technology, el cual incluye a las revistas más relevantes en el campo del laboratorio clínico, como Clinical Chemistry o Clinical Chemistry and Laboratory Medicine. Existen otros sistemas de indicadores de calidad como los de SCImago (de Elsevier) elaborados con información contenida en Scopus. Su alternativa al FI es el SCImago Journal Rank (SJR), que se calcula con un algoritmo teniendo en cuenta el número medio de citas recibidas en un año de los trabajos publicados durante los tres años anteriores ponderando la importancia de la revista que cita. Estas bases de datos, además de otras como Google Scholar Metrics, siguen desde este año a nuestra revista y le asignan una valoración.

Independiente de estos primeros números conseguidos en los rankings bibliométricos, consideramos que ha sido un gran logro llegar a este punto en un periodo de tiempo tan corto. De hecho, tanto el FI como el SJR incluyen el primer año de la revista en el cómputo de los indicadores, lo que refleja la rapidez de su inclusión. Ello se ha logrado gracias a unas grandes dosis de ilusión y esfuerzo de los que hacen la revista, al rigor y calidad exigidos desde el comienzo y, sobre todo, a la confianza que los autores han mostrado en este proyecto. También es necesario un agradecimiento especial a los revisores, cuyo trabajo desinteresado es esencial para mantener la calidad de la revista.

Una vez conseguidos estos objetivos se abren nuevos retos para que el prestigio de la revista siga creciendo a través de la publicación de buena ciencia. Ello probablemente también requerirá ser más exigentes en la calidad de los trabajos publicados y estar atentos frente a manuscritos elaborados con dudosas tácticas. Por otra parte, la calidad de la producción científica de un investigador se valora principalmente mediante el número de publicaciones en revistas que son reconocidas en base a estos indicadores. Es por ello que, indudablemente, la mayor visibilidad y el tener un FI estimularán a más autores a enviar sus trabajos a la revista. En definitiva, entramos en una nueva etapa, que sin duda ha de resultar aún más interesante que la anterior.

## References

[1] Caballé I (2020). Nace una revista para avanzar en la Medicina de Laboratorio Advances in Laboratory Medicine/Avances en Medicina de Laboratorio. Adv Lab Med.

[2] Lippi G, Mattiuzzi C, Plebani M (2015). Ranking prestige of medical and laboratory technology journals. Clin Chem Lab Med.

